# Can EGFR-TKIs be used in first line treatment for advanced non-small cell lung cancer based on selection according to clinical factors ? -- A literature-based meta-analysis

**DOI:** 10.1186/1756-8722-5-62

**Published:** 2012-10-10

**Authors:** Chongrui Xu, Qing Zhou, Yi-long Wu

**Affiliations:** 1 Guangdong Lung Cancer Institute, Guangdong General Hospital & Guangdong Academy of Medical Science, 106 Zhongshan Er Road, Guangzhou, 510080, Guangdong, P.R. China

**Keywords:** Non-small cell lung cancer, Target therapy, Chemotherapy, Meta-analysis

## Abstract

**Background:**

In the first line treatment of non-small cell lung cancer (NSCLC), several clinical trials have shown that not all NSCLC patients can benefit from treatment with tyrosine kinase inhibitors (TKIs) than receiving chemotherapy. Some trials treated patients with TKI according to their clinical characteristics. A few studies only chose patients with an epidermal grouth factor receptor (EGFR) mutation for TKI therapy. We aimed to determine whether patients could be treated with TKIs based on clinical factors in the first-line setting.

**Methods:**

We performed a meta-analysis of randomized trials involving patients with advanced NSCLC treated with chemotherapy or TKIs by different selections. Efficacy outcomes of interest were the objective response rate (ORR), progression-free survival (PFS) and the overall survival (OS) of each treatment arm.

**Results:**

Four trials enrolled unselected patients, and two trials selected East Asian patients using the clinical factors of gender and smoking history. Five trials chose patients with an EGFR mutation who were randomized for treatment with TKI or chemotherapy. For unselected patients, the risk ratio (RR) of the ORR was 3.52, the hazard ratio (HR) of the PFS was 1.29 and the HR of the OS was 1.35. For the clinically selected patients, the RR of the ORR was 0.64. The HRs of the PFS and OS were 0.83 and 0.92, respectively. The ORR and PFS were better for TKIs than for chemotherapy in patients with an EGFR mutation. The ORR was 0.47, and the HRs of the PFS and OS were 0.36 and 1.00, respectively.

**Conclusions:**

Advanced NSCLC patients with an EGFR mutation benefit most from TKIs. EGFR-TKI treatment is justified for patients with unknown EGFR status,and those who cannot tolerate chemotherapy owing to age, poor performance status (PS) or other medical conditions, when selected according to clinical factors in the first-line setting.

## Background

During the past 10 years, epidermal growth factor receptor (EGFR) tyrosine kinase inhibitors (TKIs) have become the most promising treatment for advanced non-small cell lung cancer (NSCLC). In 2003 and 2004, gefitinib and erlotinib, respectively, were approved by the U.S. Food and Drug Administration (FDA) for advanced NSCLC patients who had previously received chemotherapy. In 2005, the ISEL trial showed no benefit for patients receiving gefitinib versus placebo [1], while subgroup analysis showed a survival benefit for gefitinib-treated patients in Asia [2]. The BR.21 study, a trial focused on Caucasian patients, showed positive results for patients who received erlotinib [3].

After TKIs were shown to be more effective than a placebo treatment, several trials were performed to determine whether TKIs were superior to chemotherapy in advanced NSCLC patients [4-11]. Most trials showed that the objective response rate (ORR), progression-free survival (PFS), and the overall survival (OS) were similar between chemotherapy and TKI arms in the second-line setting. In the ISTANA and V-15-32 trials, the ORR of chemotherapy was superior to that of TKIs. When unselected NSCLC patients received TKIs in the first–line setting, the ORR, PFS, and OS were not better than those for standard chemotherapy. These results indicate that not all NSCLC patients can benefit from TKIs.

In 2004, Lynch et al. [12] and Paez et al. [13] found that patients who harbored an active mutation in EGFR derived greater benefit from TKI treatment. Several clinical trials have shown that patients with an EGFR mutation responded better and had a better PFS than patients carrying wild-type EGFR, when receiving EGFR-TKIs compared with a placebo. In 2010, two randomized trials in Japan and one randomized trial in China compared TKI treatment and chemotherapy in patients with EGFR mutations. The results confirmed that NSCLC patients with EGFR mutations can realize greater benefits from TKIs than from chemotherapy as first-line treatment. Thus, most guidelines have been updated with the consensus that an EGFR mutation is the strongest predictive factor for TKI treatment. In the clinic, however, not all NSCLC patients have adequate tissue or specimens for mutation detection, and not all patients can tolerate chemotherapy. Whether patients with unknown EGFR status should receive TKIs in the first-line setting is still controversial. EGFR-TKIs were suitable for all patients in the second-line setting, based on clinical practice. It is easy for clinicians to treat patients with TKIs according to clinical factors. Is it reasonable to choose patients for TKI treatment according to specific clinical factors?

We performed a meta-analysis of the response, PFS, and survival data between unselected, clinically selected, and EGFR mutation-selected trials to determine the best method for choosing patients who would benefit from TKI therapy in clinical practice.

## Methods

### Search strategy

The efficacy outcomes of interest were the ORR (complete and partial response) based on the Response Evaluation Criteria in Solid Tumors (RECIST), the PFS, and the OS for each treatment arm.

To find relevant articles, we searched MEDLINE, Clinical-Trials.gov, the Cochrane Library, abstracts from the World Conference for Lung Cancer (WCLC) and the annual meetings of the American Society of Clinical Oncology (ASCO) and European Society for Medical Oncology (ESMO). The medical subjects heading (MeSH) terms used for keyword and text word searching included advanced non-small cell lung cancer, gefitinib, erlotinib, and epidermal growth factor receptor (EGFR) tyrosine kinase inhibitor. In addition to computer-based searches, the reference lists in reviews and original papers were scanned to look for missing trials. No language restrictions were applied.

### Study selection

We considered all prospective, randomized, controlled trials published in peer-reviewed journals or presented at meetings of ASCO, ESMO or WCLC before 2012 in which stage IIIB/IV or post-operational recurrent NSCLC (including adenocarcinoma, squamous cell carcinoma, and large cell carcinoma) patients were prospectively randomized to receive gefitinib or erlotinib versus chemotherapy. Only articles published before December 31, 2011 were included. Whenever multiple reports of the same research were encountered, we retained only the report with the longest follow-up (largest number of events) to avoid duplication of information. We excluded dose-escalating studies, phase I or I/II trials, trials with placebo in the control arms, and studies focusing on the frequency of EGFR gene mutation and its correlation with clinical pathological status. Studies that did not clearly present ORR, PFS, or OS information were also excluded, as were studies with “triplet” chemotherapy regimens or sequential or combination therapy involving gefitinib or erlotinib with other chemotherapeutic agents.

Trials that enrolled all NSCLC patients were considered unselected trials. Trials that enrolled NSCLC patients with specific clinical characteristics such as gender, smoking history, ethnicity, or pathologic type were clinically selected trials. Trials that enrolled only patients with an EGFR mutation were EGFR mutation-selected trials.

### Statistical methods

Treatment arms with both chemotherapy and TKIs were combined regardless of dosage, regimen, or previous treatment. Patients obtaining complete response or partial response were considered as ORR. ORR data estimates of the treatment effects were obtained from the number of events reported in each arm of the trials. For time-to-event data PFS and OS, the log hazard ratios (HRs) and their variances were estimated using the methods proposed by Pignon and Hill [14]. HRs with 95% CIs were calculated using an inverse variance method.

The I-squared statistic was employed to assess variability across studies attributable to heterogeneity. Heterogeneity was considered significant at a p-value of the I-squared statistic higher than 25%. We interpreted a random-effects model because between-study heterogeneity was anticipated.

The P-values for all comparisons were two-tailed, and statistical significance was defined as P < 0.05 for all tests with the exception of those for heterogeneity. ORR of the patients was analysis of the variance with different selection. Statistical analyses were conducted with STATA 11.0.

## Results

### Eligible trials

**Figure 1:**
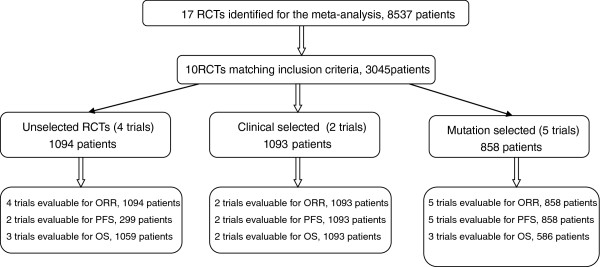
Outline of the search flow diagram.

We identified 17 randomized studies involving 8537 patients with advanced NSCLC, from computerized literature databases, reference lists of systematic reviews, and relevant articles (Figure 1). Seven trials were excluded as six used a placebo in the control arm and one combined chemotherapy with erlotinib treatment. The other 10 studies, published between 2006 and 2011, were included. The number of subjects in these studies ranged from 35 to 1466, for a total of 3045 patients. All of the studies were randomized trials with head-to-head comparisons of various chemotherapeutic regimens versus TKIs (Table 1).

**Table 1:** Characters of the eligible trials

**Author**	**Year**	**Pts**	**Treatment arm**	**Control arm**
Unselected				
Lilenbaum, R. [10]	2008	103	Erlotinib	Paclitaxel + Carboplatin
Crino, L. (INVITE) [6]	2008	196	Gefitinib	Vinorelbine
Agarwal, S. [4]	2010	35	Gefitinib	Gemcitabine + Carboplatin
Gridelli, C. (TORCH) [15]	2010	760	Erlotinib	Vinorelbine + Carboplatin
Clinical-Selected				
Lee, J. S. (First-SIGNAL) [16]	2009	313	Gefitinib	Gemcitabine + Cisplatin
Mok, T. S. (IPASS) [17,18]	2009	780	Gefitinib	Paclitaxel + Carboplatin
EGFR mutation Selected				
Mitsudomi, T. (WJTOG3405) [19]	2010	177	Gefitinib	Docetaxel + Cisplatin
Maemondo, M. (NEJSG) [20]	2010	230	Gefitinib	Paclitaxel + Carboplatin
Mok, T. S. (IPASS) [17,18]	2009	261	Gefitinib	Paclitaxel + Carboplatin
Zhou, C. (OPTIMAL) [21]	2011	165	Erlotinib	Gemcitabine + Carboplatin
Rosell, R. (EURTAC) [22]	2011	174	Erlotinib	Platium based

### Efficacy comparison

Gefitinib was the first TKI approved in non-small cell lung cancer and now is still approved in East-Asia, so several trials tried to treat patients in the first-line with Gefitinib. Trials performed in the Caucasian patients chose Erlotinib recently. We integrate the two TKIs together when performing the analysis.

Four studies of randomized NSCLC patients were based on no particular patient criteria in the first-line setting. Among them, three used gemcitabine, vinorelbine, or paclitaxel plus carboplatin. All trials reported the ORR. PFS was available for two trials; and OS for three trials.

Two trials, IPASS and First-SIGNAL, selected patients according to clinical criteria. The IPASS trial chose East Asian adenocarcinoma patients who had never smoked or were former light smokers. ORR, PFS and OS of the EGFR status unknown patients had been reported. The First-SIGNAL trial enrolled East Asian adenocarcinoma patients who had never smoked. Both trials used gefitinib as the treatment arm; the control arm was paclitaxel plus carboplatin in IPASS, and gemcitabine plus cisplatin in First-SIGNAL.

Two trials from Japan reported results in patients with an EGFR mutation randomized to receive gefitinib or chemotherapy; both trials used gefitinib as the treatment arm; docetaxel plus cisplatin and paclitaxel plus carboplatin were used in the control arms. The IPASS trial also reported the results of an EGFR mutation subgroup and was included in the meta-analysis of the EGFR mutation selection trials. Last year, the EURTAC trial reported results at the ASCO annual meeting and this was published this year. Patients with and EGFR mutation received erlotinib or platinum-based doublet. The OPTIMAL trial treated patients with an EGFR mutation randomly either with erlotinib or gemcitabine and carboplatin.

The ORR of the TKIs in the unselected, clinical selected and EGFR mutation selected was 5%, 47.7% and 69.6%, respectively. (p = 0.049)

**Figure 2:**
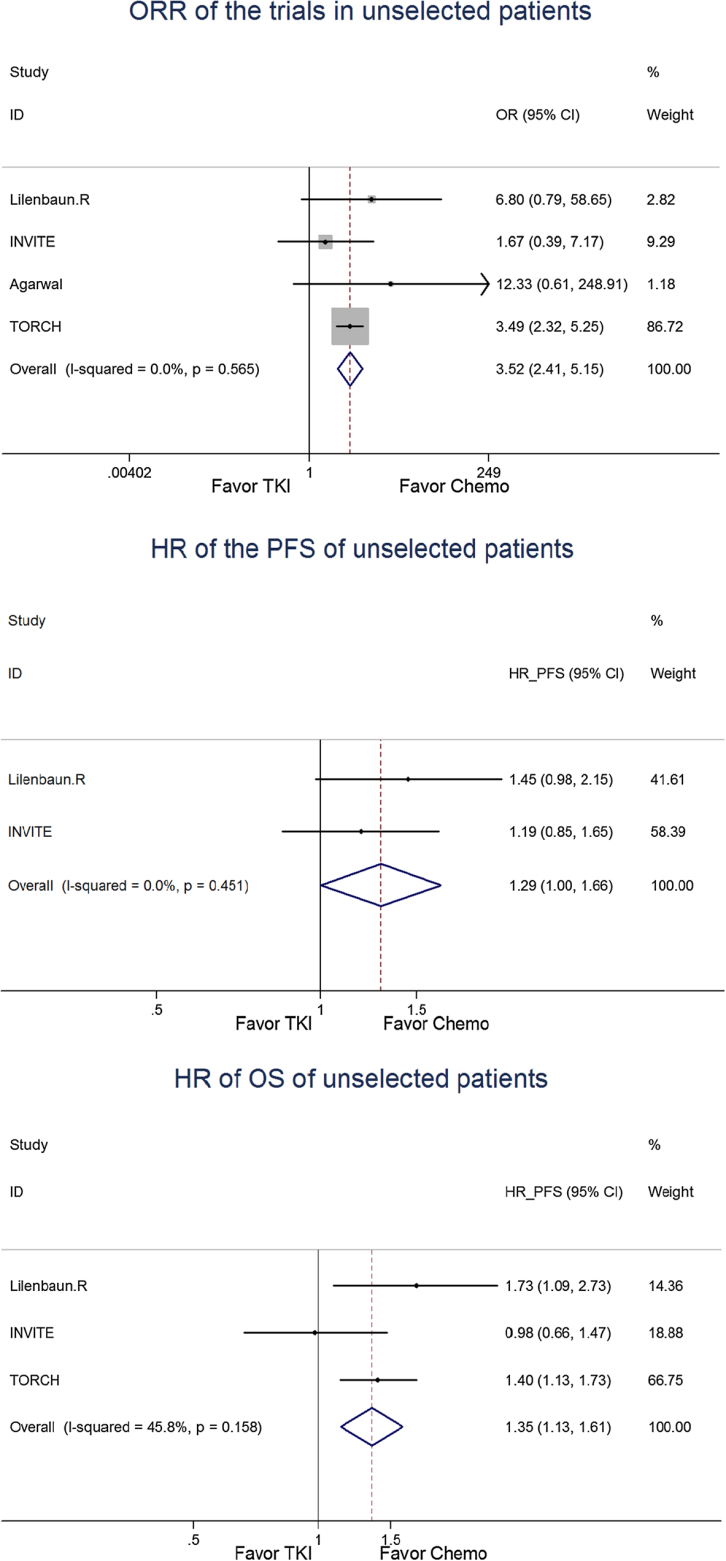
Meta-analysis of the first line trials in unselected patients.

For the unselected first-line trials, the risk ratio (RR) of the ORR was 3.52 [95% CI, 2.41–5.15] (Figure 2). The HR of the PFS was 1.29 [95% CI, 1.00–1.66] (Figure 2), and the HR of the OS was 1.35 [95% CI, 1.13–1.61] (Figure 2).

**Figure 3:**
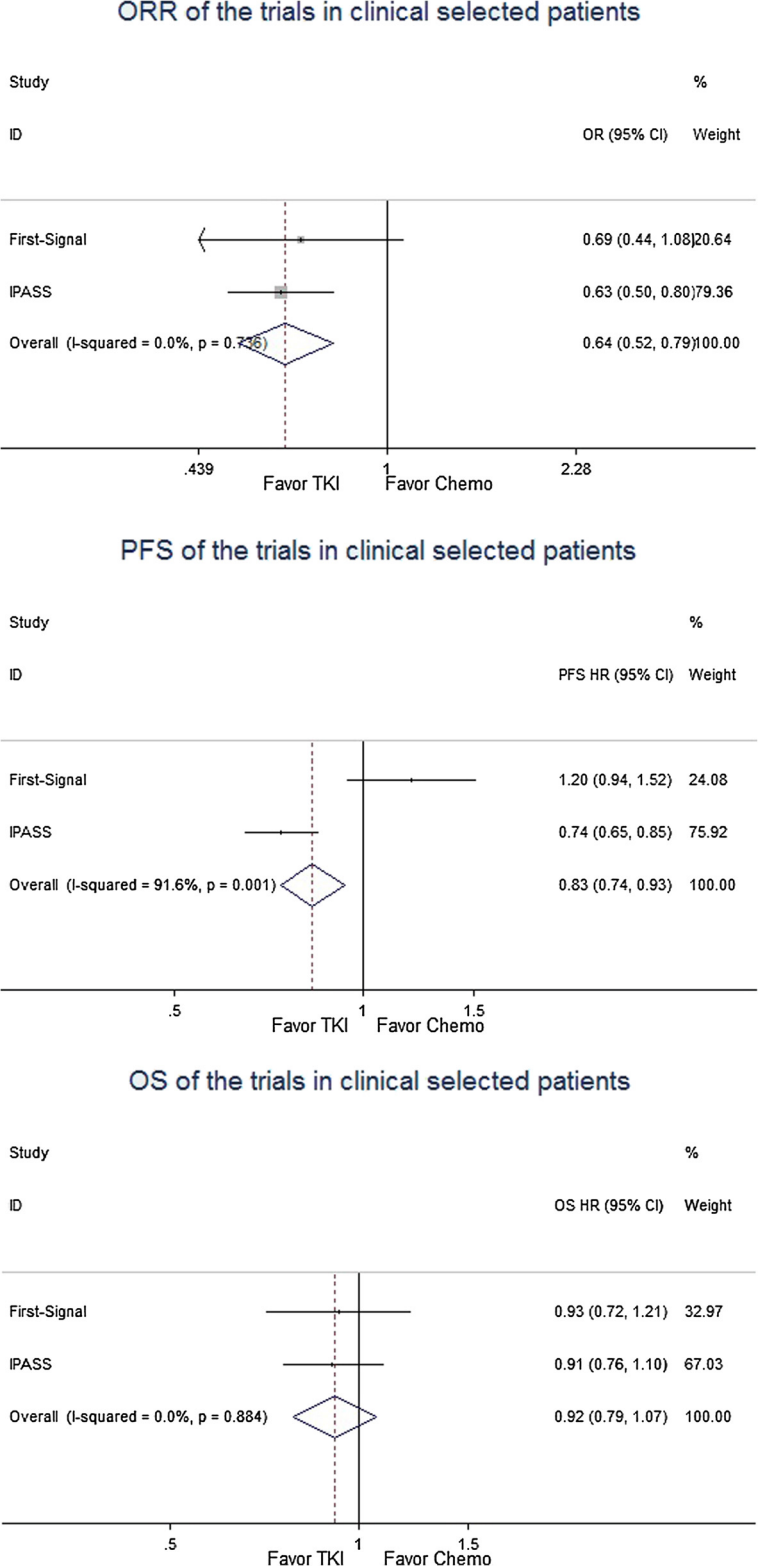
Meta-analysis of the trials in clinical selected patients with EGFR unknown status.

When patients with unknown EGFR status were chosen to receive TKIs or chemotherapy according to clinical factors, the RR of the ORR was 0.64 [95% CI, 0.52–0.79], favoring TKI (Figure 3). The HRs of the PFS and OS were 0.83 [95% CI, 0.74–0.93] (Figure 3) and 0.92 [95% CI, 0.79–1.07] (Figure 3), respectively.

**Figure 4:**
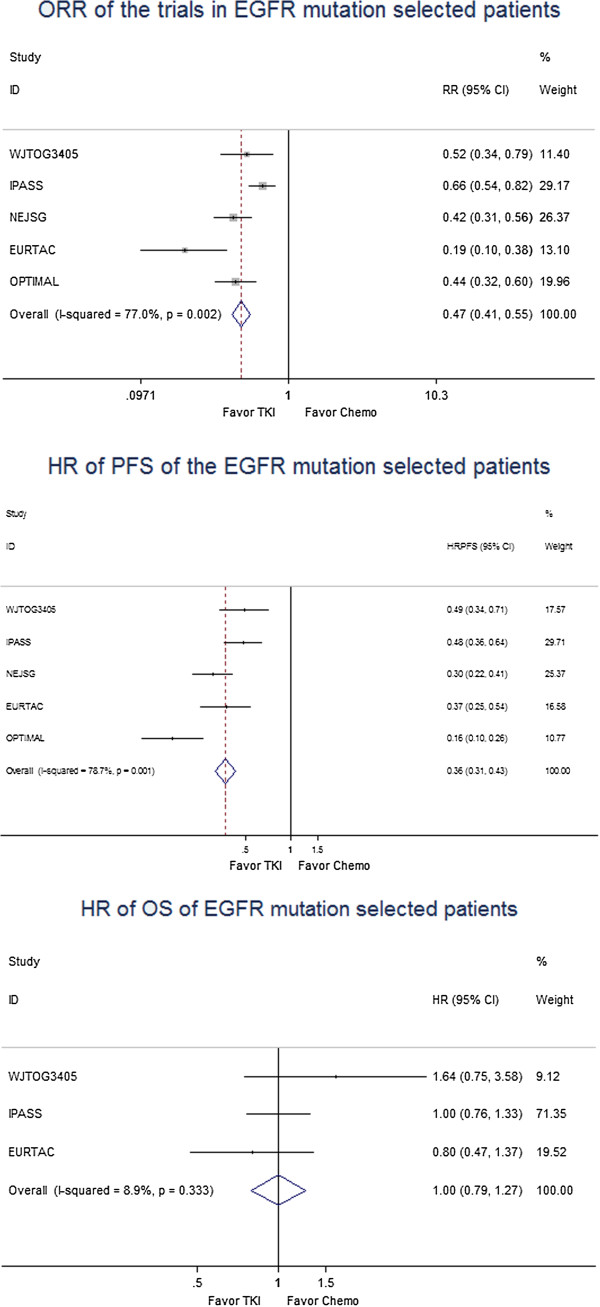
Meta-analysis of the trials in EGFR mutation selected patients.

The ORR and PFS were better for TKIs than for chemotherapy in patients with an EGFR mutation. The ORR was 0.47 [95% CI, 0.41–0.55] (Figure 4), and the HRs of the PFS and OS were 0.36 [95% CI, 0.31–0.43] (Figure 4) and 1.00 [95% CI, 0.79–1.27] (Figure 4), respectively.

## Discussion

EGFR-TKIs are the most promising development in the treatment of advanced NSCLC. Clinical trials comparing TKIs and placebos have produced controversial results. It seems that not all NSCLC patients benefit from these drugs. Different selection criteria for patients will produce different results.

The ORR of TKIs was higher in EGFR mutation selected groups than the other two groups, but it was undirected comparison. To find out how to choose TKIs or chemotherapy in different selections, TKIs versus chemotherapy was analyzed.

Four trials compared TKIs with chemotherapy in the first line setting for unselected NSCLC patients. The control arm involved standard chemotherapeutic regimens such as paclitaxel plus carboplatin or gemcitabine plus carboplatin, vinorelbine, or docetaxel. The ORR for chemotherapy was superior to the ORR for TKIs. The OS and especially the PFS tended to benefit from chemotherapy. The HR of the ORR was 3.52, and the PFS 1.29, OS 1.35 ------ all well above 1.0. These results indicate that EGFR-TKIs may be harmful to unselected patients in the first-line setting. In this situation, chemotherapy should still be used as the standard treatment for unselected patients.

Five trials reported the results of TKI treatment versus chemotherapy in patients with an EGFR mutation, showing that these patients benefitted more from TKI treatment based on both the response and PFS. The results of these five trials confirm the superiority of TKIs compared with chemotherapy for patients with an EGFR mutation. The OS for patients with an EGFR mutation was similar to chemotherapy, but there are too many factor will interfere the survival. The interference of the survival not only on the patients received TKIs in the second line but also the different chemotherapy regimen or some patients without second line therapy. The results of the meta-analysis confirmed the survival of the TKIs is similar with the chemotherapy in patients with EGFR mutations while the ORR and PFS of TKIs were dramatically superior. Patients should receive TKI treatment when their EGFR mutation status is confirmed.

In clinical practice, however, not all patients can undergo EGFR gene analysis. Salto-Tellez et al. [23] estimated that the proportion of non-squamous NSCLC patients tested for EGFR mutations varied from 30% to 80% in East Asia. Furthermore, some patients with poor performance status or with comorbidities cannot tolerate chemotherapy. Determining whether EGFR-TKIs should be used in the first-line setting for patients with unknown EGFR status and a performance status >2 is an important issue. Two trials compared TKIs and chemotherapy in patients selected according to specific clinical characteristics. Both studies were performed in East Asia and enrolled patients with an adenocarcinoma subtype and who had never smoked or were only former light smokers. A meta-analysis of the two trials showed that the patients chosen in this way had a greater response and better PFS with TKI treatment than with chemotherapy. In the TOPICAL trial [24], when all NSCLC patients with poor performance status or who were unfit for chemotherapy were given erlotinib or placebo in the first-line setting, the OS did not improve with erlotinib; however, an analysis by gender showed that the female patients benefited from erlotinib in terms of PFS and OS, while the male patients did not. Thus, in some special situations involving patients with unknown EGFR status or who are not fit for chemotherapy, a trial of EGFR-TKI may be reasonable according to clinical criteria, provided that the patient and family members have been informed of the possible worsening of symptoms and disease based on the IPASS and TORCH findings.

This meta-analysis showed that groups of advanced NSCLC patients selected according to different criteria will show differential benefits from TKIs compared with standard chemotherapy. However, this meta-analysis did not consider involving individual patient data, and only two trials performed in East Asia focused on clinical selection of patients. Most trials only report the clinical characteristics and total response of the EGFR mutation and wild type patients. The different clinical factors may have different influence on the results while this meta-analysis cannot interpret. More trials are needed involving patients with specific clinical characteristics to confirm these findings.

## Conclusions

Our meta-analysis indicates that among NSCLC patients, advanced NSCLC patients with EGFR gene mutations would benefit most from TKI treatment, especially in the first-line setting. Nevertheless, EGFR-TKI treatment is justified for patients with unknown EGFR status, those who cannot tolerate chemotherapy owing to advanced age or who have poor performance status, and those with other medical conditions, when selected according to clinical factors.

## Competing interests

The authors indicated no potential conflicts of interest.

## Authors’ contributions

YLW designed the systematic review; CX and QZ performed the literature search and extracted the data from eligible studies; CX conducted the analysis; CX, QZ, YLW were involved in the interpretation of the results. CX, QZ, YLW were responsible for the writing and critical revisions of the manuscript. All authors read and approved the final manuscript.
